# Cannabidiol Prevents Motor and Cognitive Impairments Induced by Reserpine in Rats

**DOI:** 10.3389/fphar.2016.00343

**Published:** 2016-09-28

**Authors:** Fernanda F. Peres, Raquel Levin, Mayra A. Suiama, Mariana C. Diana, Douglas A. Gouvêa, Valéria Almeida, Camila M. Santos, Lisandro Lungato, Antônio W. Zuardi, Jaime E. C. Hallak, José A. Crippa, D’Almeida Vânia, Regina H. Silva, Vanessa C. Abílio

**Affiliations:** ^1^Interdisciplinary Laboratory of Clinical Neurosciences, Department of Psychiatry, Federal University of São PauloSão Paulo, Brazil; ^2^Department of Pharmacology, Federal University of São PauloSão Paulo, Brazil; ^3^Department of Psychobiology, Federal University of São PauloSão Paulo, Brazil; ^4^Department of Neuroscience and Behavior, University of São PauloRibeirão Preto, Brazil; ^5^National Institute for Translational Medicine – National Council for Scientific and Technological DevelopmentRibeirão Preto, Brazil

**Keywords:** reserpine, cannabidiol, Parkinson’s disease, tardive dyskinesia, schizophrenia, rat

## Abstract

Cannabidiol (CBD) is a non-psychotomimetic compound from *Cannabis sativa* that presents antipsychotic, anxiolytic, anti-inflammatory, and neuroprotective effects. In Parkinson’s disease patients, CBD is able to attenuate the psychotic symptoms induced by L-DOPA and to improve quality of life. Repeated administration of reserpine in rodents induces motor impairments that are accompanied by cognitive deficits, and has been applied to model both tardive dyskinesia and Parkinson’s disease. The present study investigated whether CBD administration would attenuate reserpine-induced motor and cognitive impairments in rats. Male Wistar rats received four injections of CBD (0.5 or 5 mg/kg) or vehicle (days 2–5). On days 3 and 5, animals received also one injection of 1 mg/kg reserpine or vehicle. Locomotor activity, vacuous chewing movements, and catalepsy were assessed from day 1 to day 7. On days 8 and 9, we evaluated animals’ performance on the plus-maze discriminative avoidance task, for learning/memory assessment. CBD (0.5 and 5 mg/kg) attenuated the increase in catalepsy behavior and in oral movements – but not the decrease in locomotion – induced by reserpine. CBD (0.5 mg/kg) also ameliorated the reserpine-induced memory deficit in the discriminative avoidance task. Our data show that CBD is able to attenuate motor and cognitive impairments induced by reserpine, suggesting the use of this compound in the pharmacotherapy of Parkinson’s disease and tardive dyskinesia.

## Introduction

Alterations in the dopaminergic nigrostriatal pathway are linked to important movement disorders, such as Parkinson’s disease and tardive dyskinesia (Mehler-Wex et al., 2006). Parkinson’s disease affects 1–2% of individuals older than 60 years (Van Den Eeden et al., 2003). It comprises motor impairments (e.g., hypokinesia, tremors, muscle rigidity) and non-motor symptoms (e.g., anxiety, deficits in cognitive function; Klockgether, 2004). The pharmacotherapy of Parkinson’s disease is mainly symptomatic and is associated with important side effects, such as dyskinesia, psychosis, and abuse of anti-parkinsonian drugs (Cenci et al., 2011). Tardive dyskinesia is an extrapyramidal side effect seen in 10–30% of patients chronically treated with antipsychotics (Correll and Schenk, 2008). This prevalence increases with aging (Goldberg, 2003). Patients with tardive dyskinesia display mainly orofacial-buccal-lingual stereotypic movements, and this is irreversible in the majority of patients (Lerner et al., 2015). Therefore, advances in the pharmacotherapy of both Parkinson’s disease and tardive dyskinesia are in need. In rodents, repeated administration of reserpine induces motor impairments (e.g., catalepsy, increased oral movements, and decreased locomotor activity) accompanied by cognitive deficits. As a result, this monoamine depleting agent is used to model both Parkinson’s disease and tardive dyskinesia (Abilio et al., 2002, 2004; Silva et al., 2002; Carvalho et al., 2006; Fernandes et al., 2012; Santos et al., 2013; Leao et al., 2015; Nade et al., 2015; de Freitas et al., 2016).

Cannabidiol (CBD) is one of over 60 compounds of *Cannabis sativa*, being the most abundant after Δ^9^-tetrahydrocannabinol (Δ^9^-THC). CBD antagonizes cannabinoid CB_1_/CB_2_ receptors agonists and inhibits the reuptake of anandamide, the main endogenous cannabinoid. CBD is also an agonist of the serotonin receptor 5-HT_1A_ and of the vanilloid receptors TRPV1 and TRPV2 (Izzo et al., 2009). CBD presents antipsychotic, anxiolytic, anti-inflammatory, and neuroprotective effects (Zuardi, 2008). In Parkinson’s disease patients, treatment with CBD attenuates the psychotic symptoms induced by L-DOPA (Zuardi et al., 2009) and improves non-motor symptoms and quality of life (Chagas et al., 2014a,b). Moreover, a pre-clinical study revealed that CBD administration prevents the catalepsy induced by haloperidol, WIN 55,212-2, and L-nitro-*N*-arginine (Gomes et al., 2013). Nonetheless, so far no study has investigated CBD’s effects on the reserpine model.

The aim of this study was to investigate whether treatment with CBD would attenuate the motor and cognitive impairments induced by repeated administration of reserpine in rats.

## Materials and Methods

### Animals

Three-month-old male Wistar rats (WR; *n* = 40), from our own colony, were used. Animals were maintained in groups of five in Plexiglas cages (41 × 34 × 16.5 cm) under controlled environmental conditions (22–23°C, light/dark cycle: lights on 6:30–18:30) with free access to food and water. The procedures of the present study were approved by the Ethics Committee of Federal University of São Paulo (N° 7798280515), and followed the guidelines of the Committee on Care and Use of Laboratory Animal Resources, National Research Council, USA, and of the Brazilian law for the use of animals in research (Law Number 11.794). All animals were drug-naïve.

### Drugs

Cannabidiol (THC-Pharm, Frankfurt, Germany) was prepared daily, diluted in saline and 1% tween-80. Reserpine (Sigma Chemical Co., St. Louis, MO, USA) was diluted in 0.5% glacial acetic acid and distilled water. CBD and its vehicle were administered intraperitoneally. Reserpine and its vehicle were administered subcutaneously. Solutions were given in a volume of 1 ml/kg of body weight.

### Behavioral Analysis

#### Locomotor Activity

Locomotor activity (Abilio et al., 2003b) was assessed in a circular open-field arena (97 cm in diameter and 32.5 cm high, with an open top and a floor divided into 19 similar quadrants). The animals were individually placed on the apparatus. Number of floor squares entered was quantified by an automated activity monitoring system (AnyMaze, Stoelting, USA) during 5 min.

#### Catalepsy

Catalepsy behavior (Fernandes et al., 2012) was assessed by placing each animal’s forepaws on a horizontal bar elevated 9 cm from the bench surface. The amount of time the animal remained in the same imposed position was scored live until a maximum of 180 s. Three trials were carried out for each animal in each observational day. The results were analyzed considering the mean value of the three trials.

#### Vacuous Chewing Movements

Vacuous chewing movements (Abilio et al., 2004) were assessed by placing the animals in individual wired cages (40 × 40.5 × 20 cm). Mirrors were placed behind the back and under the bottom to allow behavioral quantification when the animal faced away from the observer. The amount of vacuous chewing movements (mouth openings in the vertical plane not directed toward physical material) was quantified live during 10 min.

#### Plus-Maze Discriminative Avoidance Task

Plus-maze discriminative avoidance task allows the simultaneous evaluation of learning, memory, anxiety, and locomotor activity (Fernandes et al., 2012). The apparatus employed was a modified elevated plus-maze, comprising two enclosed arms (50 × 15 × 40 cm) opposite to two open arms (50 × 15 cm). A lamp and a speaker were placed over one of the enclosed arms (aversive arm). In the training session, each animal was placed in the center of the apparatus. Every time it entered the aversive enclosed arm, the animal was submitted to 100 W light and 80 dB noise (aversive stimuli) until it left the arm. Twenty-four hours later, the animals were submitted to the test session. Each animal was placed in the center of the apparatus without being submitted to aversive stimuli (the lamp and the speaker were still placed over the aversive arm, but were not turned on). Both sessions lasted 10 min.

Distance traveled in the apparatus (used for evaluation of motor activity) and time spent in each arm (aversive, non-aversive, and open arms) were quantified in training and test sessions at 1-minute intervals by an automated activity monitoring system (AnyMaze, Stoelting, USA). Percent time in the open arms (total time spent in open arms/total time spent in open and enclosed arms) in training session was calculated to evaluate anxiety. Percentage of time in the aversive arm (time spent in aversive enclosed arm/time spent in both enclosed arms), assessed minute by minute across the training session, was employed to assess learning. Total time spent in the aversive vs. non-aversive arms in the training session was also used to evaluate learning. Total time spent in the aversive vs. non-aversive arms in the test session was used to evaluate memory.

The observers were blind to the animals’ experimental condition.

### Experimental Design

**FIGURE 1:**
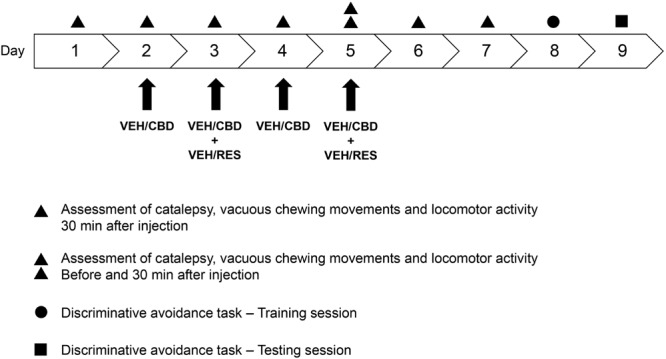
**Experimental design.** The animals received four injections of CBD (0.5 or 5 mg/kg) or vehicle (saline + 1% tween-80) on days 2–5, and two injections of reserpine (1 mg/kg) or vehicle (0.5% glacial acetic acid + distilled water) on days 3 and 5. Locomotor activity, vacuous chewing movements, and catalepsy behavior were assessed on days 1–7. On days 8 and 9, animals were submitted to the training and test sessions of the discriminative avoidance task, respectively. CBD, cannabidiol; RES, reserpine; VEH, vehicle. *n* = 10/group.

The animals received four injections of CBD (0.5 or 5 mg/kg) or vehicle on days 2–5, and two injections of reserpine (1 mg/kg) or vehicle on days 3 and 5. The drug regimen for the administration of reserpine was based on previous reports that show reserpine-induced oral dyskinesia, catalepsy, and memory impairments (Silva et al., 2002; Abilio et al., 2003a, 2004). The doses of CBD were chosen based on a previous work from our group showing beneficial effects of CBD at this same range of doses (Levin et al., 2012). Locomotor activity, vacuous chewing movements, and catalepsy behavior were assessed on days 1–7. On days 8 and 9, animals were submitted to the training and test sessions of the discriminative avoidance task, respectively (**Figure 1**).

The experimental groups were: vehicle-vehicle (control group), vehicle-reserpine, CBD 0.5-reserpine, and CBD 5-reserpine (*n* = 10/group).

### Statistical Analysis

Data from catalepsy, vacuous chewing movements, locomotor activity and percentage of time spent in the open arms in the training session of the discriminative avoidance task were analyzed by one-way ANOVA, followed by Bonferroni’s *post hoc*.

Comparison between total time spent in the aversive- and non-aversive enclosed arms on the training and test sessions of discriminative avoidance task was analyzed by paired-samples *t*-test.

Data from the percent time spent in the aversive enclosed arm at 1-minute intervals throughout the training session were analyzed by repeated measures ANOVA, with time within session as the within-subject factor.

## Results

### Locomotor Activity

One-way ANOVA revealed effect of treatment on days 5 (after injection) [*F*(3,39) = 3.694; *p* < 0.05], 6 [*F*(3,39) = 6.030; *p* < 0.005] and 7 [*F*(3,39) = 6.627; *p* < 0.005]. Bonferroni’s test showed that on day 5 (after injection), the group vehicle-reserpine displayed decreased locomotion when compared to the control group (vehicle-vehicle). On day 6, all the reserpine-treated groups displayed diminished locomotor activity when compared to control. On day 7, vehicle-reserpine and CBD 0.5-reserpine groups, but not CBD 5-reserpine, displayed decreased locomotor activity when compared to control. In all comparisons, CBD-treated groups did not differ from vehicle-reserpine group. Treatment with CBD was not able to attenuate the decrease in locomotion induced by reserpine (**Table 1**).

**Table 1:** Squares crossed in the open field arena by Wistar rats (*n* = 10/group) treated with CBD (0.5 or 5 mg/kg) or vehicle (VEH – saline + 1% tween-80) in addition to reserpine (RES – 1 mg/kg) or vehicle (VEH – 0.5% glacial acetic acid + distilled water).

	VEH-VEH	VEH-RES	CBD 0.5-RES	CBD 5-RES
Day 1	82.9 ± 5.9	93.0 ± 8.9	98.2 ± 5.0	108.0 ± 7.0
Day 2	78.7 ± 9.2	85.5 ± 12.8	78.3 ± 7.8	89.9 ± 7.6
Day 3	72.6 ± 8.9	63.9 ± 14.2	62.1 ± 10.9	64.2 ± 10.5
Day 4	44.7 ± 10.8	29.2 ± 9.7	16.8 ± 4.8	23.0 ± 4.4
Day 5 Before	36.4 ± 10.3	31.5 ± 9.5	15.5 ± 4.2	28.1 ± 12,3
Day 5 After	31.8 ± 6.0	5.3 ± 1.9^∗^	14.8 ± 6.5	13.0 ± 6.8
Day 6	40.4 ± 10.5	12.9 ± 5.2^∗^	10.7 ± 3.2^∗^	7.4 ± 2.2^∗^
Day 7	46.4 ± 9.0	13.1 ± 2.5^∗^	16.5 ± 4.5^∗^	24.0 ± 5.3

*^∗^*p* < 0.05 when compared to VEH-VEH group. One-way ANOVA followed by Bonferroni’s test. Data expressed as mean ± SEM.*

### Catalepsy

**FIGURE 2:**
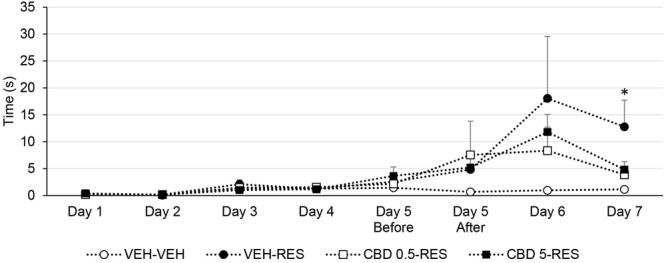
**Catalepsy time of Wistar rats (*n* = 10/group) treated with CBD (0.5 or 5 mg/kg) or vehicle (VEH – saline + 1% tween-80) in addition to reserpine (RES – 1 mg/kg) or vehicle (VEH – 0.5% glacial acetic acid + distilled water).** ^∗^*p* < 0.05 when compared to VEH-VEH group. One-way ANOVA followed by Bonferroni’s test. Data expressed as mean ± SEM.

One-way ANOVA revealed effect of treatment on day 7 [*F*(3,39) = 3.510; *p* < 0.05]. Bonferroni’s test showed that vehicle-reserpine group displayed increased catalepsy behavior when compared to control group. CBD-treated groups did not differ from control or from vehicle-reserpine group. Treatment with CBD attenuated the reserpine-induced increase in catalepsy (**Figure 2**).

### Vacuous Chewing Movements

**FIGURE 3:**
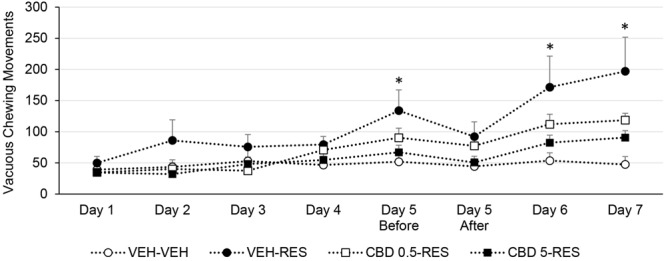
**Vacuous chewing movements of Wistar rats (*n* = 10/group) treated with CBD (0.5 or 5 mg/kg) or vehicle (VEH – saline + 1% tween-80) in addition to reserpine (RES – 1 mg/kg) or vehicle (VEH – 0.5% glacial acetic acid + distilled water).** ^∗^*p* < 0.05 when compared to VEH-VEH group. One-way ANOVA followed by Bonferroni’s test. Data expressed as mean ± SEM.

One-way ANOVA revealed effect of treatment on days 5 (before injection) [*F*(3,39) = 3.069; *p* < 0.05], 6 [*F*(3,39) = 3.332; *p* < 0.05], and 7 [*F*(3,39) = 4.661; *p* < 0.05]. Bonferroni’s test showed that vehicle-reserpine group displayed increased vacuous chewing movements when compared to control group. CBD-treated groups did not differ from control or from vehicle-reserpine group. Treatment with CBD attenuated the reserpine-induced increase in oral movements (**Figure 3**).

### Discriminative Avoidance Task

**FIGURE 4:**
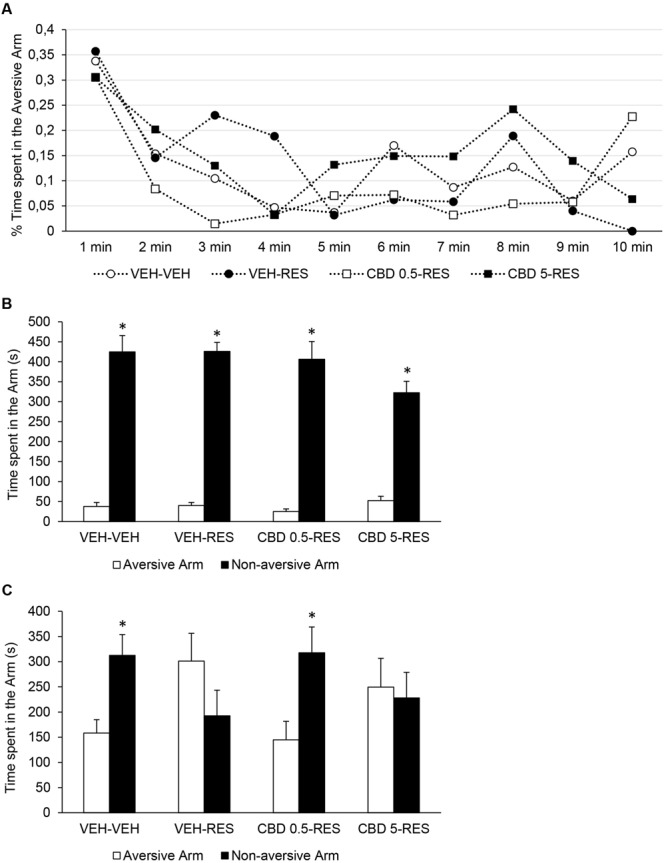
**Performance in the discriminative avoidance task of Wistar rats (*n* = 10/group) treated with CBD (0.5 or 5 mg/kg) or vehicle (VEH – saline + 1% tween-80) in addition to reserpine (RES – 1 mg/kg) or vehicle (VEH – 0.5% glacial acetic acid + distilled water).** Percentage of time spent in the aversive arm in the training session at 1-minute intervals **(A)**. Time spent in aversive and non-aversive enclosed arms in the training **(B)** and test **(C)** sessions. ^∗^*p* < 0.05 when compared to the aversive arm. Repeated measures ANOVA **(A)** and paired-samples *t*-test **(B,C)**. Data expressed as mean ± SEM.

For the percent time spent in the aversive arm in the training session, repeated one-way ANOVA revealed effect of time [*F*(9,315) = 7.351; *p* < 0.001], but not of treatment or of an interaction between factors. All groups showed a decrease in the time spent in the aversive arm throughout the session (**Figure 4A**). In addition, paired *t*-test showed that all groups spent more time in the non-aversive enclosed arm than in the aversive enclosed arm: vehicle-vehicle [*t*(9) = -7.667; *p* < 0.001], vehicle-reserpine [*t*(9) = -13.900; *p* < 0.001], CBD 0.5-reserpine [*t*(9) = -8.984; *p* < 0.001], and CBD 5-reserpine [*t*(9) = -8.321; *p* < 0.001] (**Figure 4B**). All groups showed adequate learning of the task.

In the test session, paired *t*-test showed that the time spent in the aversive enclosed arm is lower than the time spent in the non-aversive enclosed arms for vehicle-vehicle [*t*(9) = -2.366; *p* < 0.05] and CBD 0.5-reserpine [*t*(9) = -2.289; *p* < 0.05] groups, but not for the vehicle-reserpine and CBD 5-reserpine groups. Treatment with reserpine impaired animals’ retrieval of the discriminative avoidance task, and this effect was prevented by treatment with CBD 0.5, but not with CBD 5 (**Figure 4C**).

Moreover, in the training session there was no effect of treatment on the percent time spent in the open arms, but treatment affected the distance traveled [*F*(3,39) = 6.362; *p* < 0.001] (**Table 2**). All groups treated with reserpine displayed diminished locomotor activity when compared to control group, and treatment with CBD and/or reserpine did not alter animals’ anxiety levels.

**Table 2:** Distance traveled (m) and percentage of time spent in the open arms in the training session of the plus-maze discriminative avoidance task by Wistar rats (*n* = 10/group) treated with CBD (0.5 or 5 mg/kg) or vehicle (VEH – saline + 1% tween-80) in addition to reserpine (RES – 1 mg/kg) or vehicle (VEH – 0.5% glacial acetic acid + distilled water).

	Distance traveled (m)	% Time spent in open arms
VEH-VEH	25.5 ± 4.9	14.4 ± 3.4
VEH-RES	12.2 ± 1.8^∗^	5.9 ± 1.7
CBD 0.5-RES	12.4 ± 1.1^∗^	9.4 ± 4.3
CBD 5-RES	10.6 ± 1.2^∗^	8.3 ± 3.1

*^∗^*p* < 0.05 when compared to VEH-VEH group. One-way ANOVA followed by Bonferroni’s test. Data expressed as mean ± SEM.*

## Discussion

The present study showed that CBD administration ameliorates motor and cognitive impairments promoted by reserpine, an animal model for both Parkinson’s disease and tardive dyskinesia. CBD attenuated the reserpine-induced catalepsy behavior, increase in oral movements and memory deficit, but not the decrease in locomotor activity. In addition, treatment with CBD and/or reserpine did not modify animals’ anxiety levels.

Reserpine irreversibly blocks the vesicular monoamine transporters 1 and 2 (VMAT-1 and VMAT-2), interfering with the storage of monoamines in synaptic vesicles. As a consequence, it increases the intracellular levels of monoamines and induces monoamine depletion in nerve terminals. The increase in intracellular levels of monoamines enhances their metabolism by the enzyme monoamine oxidase (MAO), increasing oxidative stress (for a review, see Leao et al., 2015). Here, reserpine administration augmented rats’ oral movements – a model for tardive dyskinesia (Waddington et al., 1983; Neisewander et al., 1994). Reserpine also increased rats’ catalepsy behavior and decreased their locomotor activity – motor impairments used to model symptoms of Parkinson’s disease (Leao et al., 2015). These effects are in accordance with previous reports (Abilio et al., 2002, 2004; Silva et al., 2002; Carvalho et al., 2006; Fernandes et al., 2012; Santos et al., 2013; Leao et al., 2015; Nade et al., 2015; de Freitas et al., 2016).

It is noteworthy that some schizophrenia patients display both parkinsonism-like symptoms and tardive dyskinesia (Fann and Lake, 1974; Richardson and Craig, 1982; Bitton and Melamed, 1984; Saito et al., 1986). This coexistence indicates that these motor disturbances share a common pathophysiological mechanism. Indeed, oxidative damage – a widely described outcome of reserpine administration (Abilio et al., 2002, 2003a; Burger et al., 2003; Fernandes et al., 2012; Nade et al., 2013; Leao et al., 2015) – is linked to both Parkinson’s disease and tardive dyskinesia. Augmented oxidative stress indices are seen in schizophrenia patients with tardive dyskinesia, when compared to those without it (Zhang et al., 2007). Regarding Parkinson’s disease, several studies show increased oxidative stress parameters in peripheral blood, cerebrospinal fluid, and brains of patients (Dexter et al., 1994; Serra et al., 2001; Boll et al., 2008; Sharma et al., 2008; Mythri et al., 2011; de Farias et al., 2016). Also, the genetic defects associated with Parkinson’s disease are directly or indirectly related to oxidative stress (Gao and Hong, 2011).

Interestingly, reserpine effects on locomotor activity and vacuous chewing movements were not seen until day 5 (second injection of reserpine) and the effect on catalepsy was only statistically significant on day 7 (48 h after the second injection of reserpine). Also, the effect of reserpine on memory was observed 96 h after the second injection. The time course for the emergence of motor abnormalities and memory impairments suggests a non-acute progressive effect of reserpine. Therefore, this effect fits better to the oxidative damage than to the acute monoamine depletion induced by reserpine.

Cannabidiol (0.5 or 5 mg/kg) attenuated the increase in catalepsy behavior and in oral dyskinesia, which is in accordance with clinical and pre-clinical findings. In humans, CBD is able to improve dystonia (Consroe et al., 1986; Sandyk et al., 1986). Parkinson’s disease patients treated with CBD during 4 weeks, in addition to their usual treatment, show a decrease in the score of the Unified Parkinson’s Disease Rating Scale (UPDRS) that assess motor and non-motor symptoms (Zuardi et al., 2009). In mice, CBD acute administration prevents the increase in catalepsy behavior induced by haloperidol (an antagonist of dopamine D_2_ receptors), WIN 55,212-2 (a CB_1_/CB_2_ agonist), and L-nitro-*N*-arginine (a non-selective inhibitor of nitric oxide synthase), therefore showing CBD’s potential on inhibiting the catalepsy induced by different mechanisms of action (Gomes et al., 2013).

Conversely, CBD was not able to attenuate – only to delay – the reserpine-induced decrease in locomotor activity, suggesting it is effective in inhibiting the emergence of some but not all the parkinsonism-like motor abnormalities. This outcome also indicates that the pathophysiological mechanisms related to the reserpine effect on locomotion are distinct from those on catalepsy and oral movements. In fact, while catalepsy behavior and oral movements in rats are linked to the dopaminergic nigrostriatal pathway (Morelli et al., 1981; Carey, 1983; Neisewander et al., 1996), locomotor activity is associated with the mesolimbic pathway (Kelly and Iversen, 1976; Carey, 1983; Neisewander et al., 1996). Interestingly, when administered intra-nucleus accumbens shell, CBD presents an antipsychotic action against amphetamine-induced locomotor sensitization and deficit in prepulse inhibition of startle. These effects seem to be mediated by different striatal molecular mechanisms than those of the known antipsychotic drugs. The authors suggest that this differential molecular signaling might be related to CBD’s absence of adverse effects typically associated with antipsychotics (Renard et al., 2016). In regard to motor side effects, this rationale suits the beneficial effects of CBD against the motor impairments induced by reserpine.

Reserpine did not alter anxiety-like behavior or learning, but impaired memory in the discriminative avoidance task. These results are in accordance with previous reports using rats and mice (Silva et al., 2002; Carvalho et al., 2006; Fernandes et al., 2008). Cognitive impairments are an important feature of Parkinson’s disease and are associated with poorer quality of life (Lawson et al., 2016). Regarding tardive dyskinesia, schizophrenia patients with this motor abnormality display more severe cognitive impairment (Waddington and Youssef, 1996; Wu et al., 2013, 2014; Fervaha et al., 2015). Our results demonstrate that CBD is able to attenuate the reserpine-induced memory deficit without modifying animals’ locomotor activity or anxiety-like behavior. Although no study has investigated the effects of CBD on the reserpine model, our data are in agreement with reports showing beneficial effects of CBD on cognitive impairments in animal models of schizophrenia (Levin et al., 2012), cerebral malaria (Campos et al., 2015), pneumococcal meningitis (Barichello et al., 2012), hepatic encephalopathy (Magen et al., 2009), and neurodegenerative disorders (Fagherazzi et al., 2012). Considering the role of the mesolimbic pathway on cognitive processes (Pezze and Feldon, 2004; Rinaldi et al., 2012; Braun et al., 2016), it is worth mentioning that intra-accumbal administration of CBD modulates an emotional memory task (Norris et al., 2016).

It is worth mentioning that only the 0.5 mg/kg dose of CBD had a beneficial effect on memory. This is in agreement with previous data showing an inverted U-shaped dose-response curve for CBD on behavioral assessments. The CBD inverted U-shaped curve is seen in pre-clinical tests of anxiety and prepulse inhibition of startle (Guimaraes et al., 1990; Levin et al., 2014; Nazario et al., 2015). In addition, studies from our group show that only the lowest dose of CBD (1 mg/kg) is effective against the social interaction and contextual fear conditioning deficits in an animal model of schizophrenia (Levin et al., 2012; Almeida et al., 2013). Inverted U-shaped dose-response curves are also seen for other cannabinoid drugs, such as WIN 55,212-2 (Almeida et al., 2014; Levin et al., 2014) and Δ^9^-THC (El-Alfy et al., 2010). This U-shaped pattern is probably the result of the modulatory role of the endocannabinoid system on different neurotransmission targets.

The mechanisms whereby CBD exerts these beneficial effects are beyond the scope of this work. However, as mentioned, oxidative stress is linked to the reserpine effects, to Parkinson’s disease and tardive dyskinesia. Previous data from our group show that the effects of reserpine are potentiated by a pro-oxidant compound (Calvente et al., 2002) and attenuated by the free radical scavengers vitamin C, vitamin E, and melatonin (Abilio et al., 2002, 2003a; Faria et al., 2005). In accordance, CBD has been described to present antioxidant, anti-inflammatory, and neuroprotective actions. The antioxidant effects of CBD are seen in rat models of binge alcohol consumption (Hamelink et al., 2005), sepsis (Cassol-Jr et al., 2010), mania (Valvassori et al., 2011), epilepsy (Hosseinzadeh et al., 2016), and Huntington (Sagredo et al., 2011). In the 6-hydroxydopamine rat model for Parkinson’s disease, treatment with CBD for 2 weeks prevents the neurodegeneration produced by the unilateral injection of the toxin into the medial forebrain bundle (Lastres-Becker et al., 2005). CBD administration in this model up-regulates the mRNA levels for the antioxidant enzyme copper-zinc superoxide dismutase (Garcia-Arencibia et al., 2007). Although other mechanisms cannot be disregarded, CBD’s antioxidant and anti-inflammatory actions are possibly involved in its beneficial effects on the reserpine model.

Aiming to diminish the amount of animals used in the study, we did not include groups treated with CBD and vehicle. Nonetheless, multiple studies show that CBD does not induce catalepsy or oral dyskinesia *per se*, even at high doses (Zuardi et al., 1991; Moreira and Guimaraes, 2005; Hayakawa et al., 2008; Long et al., 2010; Gomes et al., 2013; Dos-Santos-Pereira et al., 2016). Studies also report absence of CBD’s effect on locomotor activity in the dose range used here (Hayakawa et al., 2008; Long et al., 2010; Almeida et al., 2013).

In summary, we showed here that CBD can attenuate the motor and cognitive impairments induced by reserpine. These data suggest CBD’s application on the treatment of tardive dyskinesia and Parkinson’s disease, conditions whose pharmacotherapy remain unsatisfactory. Therefore, preventing the emergence of motor symptoms would represent a major advance in patients’ quality of life. It is worth mentioning that CBD is also effective on treating the psychotic symptoms of both Parkinson’s disease and schizophrenia patients (Iseger and Bossong, 2015) without inducing the parkinsonian and dyskinetic adverse effects associated with classical antipsychotic drugs. Further studies are in need, but data suggest that including CBD on the pharmacotherapy of Parkinson’s disease and tardive dyskinesia might be beneficial to the motor and cognitive impairments, and also to patients’ psychiatric symptoms.

## Author Contributions

FP, RL, and VCA designed the study. FP, RL, MS, MD, DG, VA, CS, and LL conducted the behavioral experiments and the statistical analysis. FP and VCA wrote the first draft of the manuscript. All authors contributed to and have approved the final manuscript.

## Conflict of Interest Statement

JH, AZ, and JC are co-inventors (Mechoulam R, Crippa JA, Guimaraes FS, Zuardi AW, Hallak JE, Breuer A.) of the patent “Fluorinated CBD compounds, compositions and uses thereof. Pub. No.: WO/2014/108899. International Application No.: PCT/IL2014/050023”; Def. US no. Reg. 62193296; 29/07/2015; INPI em 19/08/2015 (BR1120150164927). University of São Paulo licensed it to Phytecs Pharm (Resolução USP No. 15.1.130002.1.1). University of São Paulo has an agreement with Prati-Donaduzzi (Toledo, Brazil): “Desenvolvimento de um produto farmacêutico contendo canabidiol sintético e comprovação de sua segurança e eficáacia terapêutica na epilepsia, esquizofrenia, doença de Parkinson e transtornos de ansiedade”. JC received a BSPG-Pharm travel Grant award. The other authors declare that the research was conducted in the absence of any commercial or financial relationships that could be construed as a potential conflict of interest.
